# ﻿A new species of the bandfish genus *Owstonia* Tanaka, 1908 (Perciformes, Cepolidae) from Taiwan, northwestern Pacific Ocean, with new records and comments on morphological characters of congeners

**DOI:** 10.3897/zookeys.1262.167436

**Published:** 2025-12-03

**Authors:** Yo Su, Hsuan-Ching Ho

**Affiliations:** 1 International Doctoral Program of Marine Science and Technology, National Sun Yat-sen University, Kaohsiung, Taiwan National Kaohsiung University of Science and Technology Kaohsiung Taiwan; 2 Department and Graduate Institute of Aquaculture, National Kaohsiung University of Science and Technology, Kaohsiung, Taiwan National Sun Yat-sen University Kaohsiung Taiwan; 3 Taiwan Ocean Research Institute, National Applied Research Laboratories, Kaohsiung, Taiwan Taiwan Ocean Research Institute, National Applied Research Laboratories Kaohsiung Taiwan; 4 National Museum of Marine Biology and Aquarium, Pingtung, Taiwan National Museum of Marine Biology and Aquarium Pingtung Taiwan; 5 Australian Museum, Sydney, Australia Australian Museum Sydney Australia

**Keywords:** Actinopterygii, biodiversity, ichthyology, morphology, new record

## Abstract

The bandfish genus *Owstonia* Tanaka, 1908 shows high diversity, with many new species described recently by Smith-Vaniz and Johnson (2016). However, not many Taiwanese materials were included, and the diversity remains largely unknown. In this study, we investigated specimens deposited in the National Museum of Marine Biology and Aquarium, which revealed that those previously reported as *O. psilos* includes *O. doryptera* (Fowler, 1934) and a species newly described here; one of three specimens reported as *O totomiensis* is re-identified as O. cf. aurora. The specimens of *O. doryptera* re­present the first record from Taiwan, as well as the northernmost record of the species. Detailed descriptions of each species are provided, with comments on their morphological variations. Moreover, the diagnostic characters proposed by previous literature are discussed. Lastly, a checklist of the species of *Owstonia* from Taiwan is provided and discussed. In total, nine species are recognized in waters of Taiwan.

## ﻿Introduction

The bandfish genus *Owstonia* Tanaka, 1908 is the most diverse genus within the family Cepolidae, with 37 species currently recognized as valid (Smith-Vaniz and Johnson 2016; Liao et al. 2022). *Owstonia* differs from the other two genera of cepolids, *Cepola* Linnaeus, 1764 and *Acanthocepola* Bleeker, 1874, in having: dorsal-fin rays III–IV, 19–26; anal-fin rays I–II, 11–19; total vertebrae 27–33; last soft rays of dorsal and anal fins not connected to caudal fin via membrane, and last pterygiophore of dorsal and anal fins support two soft rays (Smith-Vaniz 2001; Smith-Vaniz and Johnson 2016).

The diversity of *Owstonia* was previously underestimated until Smith-Vaniz and Johnson (2016) conducted a comprehensive review of the genus and described 21 new species. However, their study included few specimens from Taiwan, leaving the full extent of species composition in the region unclear. Previous literature included four species from Taiwan: *O. grammodon* (Fowler, 1934), *O. taeniosoma* (Kamohara, 1935), *O. tosaensis* (Kamohara, 1934), and *O. totomiensis* (Tanaka, 1908) (Shen 1993; Shen and Wu 2011; Smith-Vaniz and Johnson 2016). Recently, Jhan and Ho (2019) newly reported five species from southwestern Taiwan, including *O. kamoharai* Endo, Liao & Mastuura, 2015 and *O. psilos* Smith-Vaniz & Johnson, 2016 as new to Taiwan. They also noted that the vertebral formula of *O. kamoharai* differs from that of type series (12 + 15 vs 11 + 17–18 in type series; Endo et al. 2015).

Recent collections and identifications of specimens of *Owstonia* by the first author revealed several inconsistencies in literature records, which led us to further re-examine the majority of specimens of *Owstonia* deposited at the National Museum of Marine Biology and Aquarium (NMMB-P), including those previously documented by Jhan and Ho (2019). Our review revealed that the specimens reported as *O. psilos* include two distinct species: *O. doryptera* (Fowler, 1934) and *O. smithvanizi* sp. nov. Moreover, one of the specimens of *O totomiensis* reported by Jhan and Ho (2019) is re-identified as O. cf. aurora.

Detailed descriptions are provided, with discussion on the diagnostic characters proposed by Smith-Vaniz and Johnson (2016). Additionally, a checklist of species of *Owstonia* from Taiwan is provided and discussed.

## ﻿Materials and methods

Specimens were fixed in 4% formaldehyde and transferred to 70% ethanol for permanent preservation. The specimens were deposited at the Pisces Collection, National Museum of Marine Biology and Aquarium, Pingtung, Taiwan (**NMMB-P**).

Terminology and methodology follow Smith-Vaniz and Johnson (2016), with the following additions: number of pseudobranchial filaments, pseudobranchial filaments counted from the inner face of right operculum; dorsal and anal-fin heights, longest soft ray of dorsal and anal fins; interorbital width, least distance of interorbital space; pectoral-fin length, from upper end of base to posterior-most tip of fin ray; pelvic-fin spine length, from origin of pelvic fin to tip of pelvic-fin spine. Paired characters are presented as left/right whenever available. The number of gill rakers at angle was included in the lower-raker count.

Vertebral formulae were determined by a digital x-ray machine at NMMBA. Measurements were made with calipers and rounding to the nearest 0.1 mm. Morphometric data were presented as percentages of standard length (SL) and/or head length (HL), unless otherwise stated.

## ﻿Results

### ﻿Family Cepolidae

#### ﻿ Owstonia smithvanizi sp. nov.

Taxon classificationAnimaliaTeleosteiCepolidae

8C6C0704-F2DE-57E4-92F1-8BEDDAD233BD

https://zoobank.org/EAF827DC-1604-40A0-838C-F0FF66B0128C

**Figure 1.**
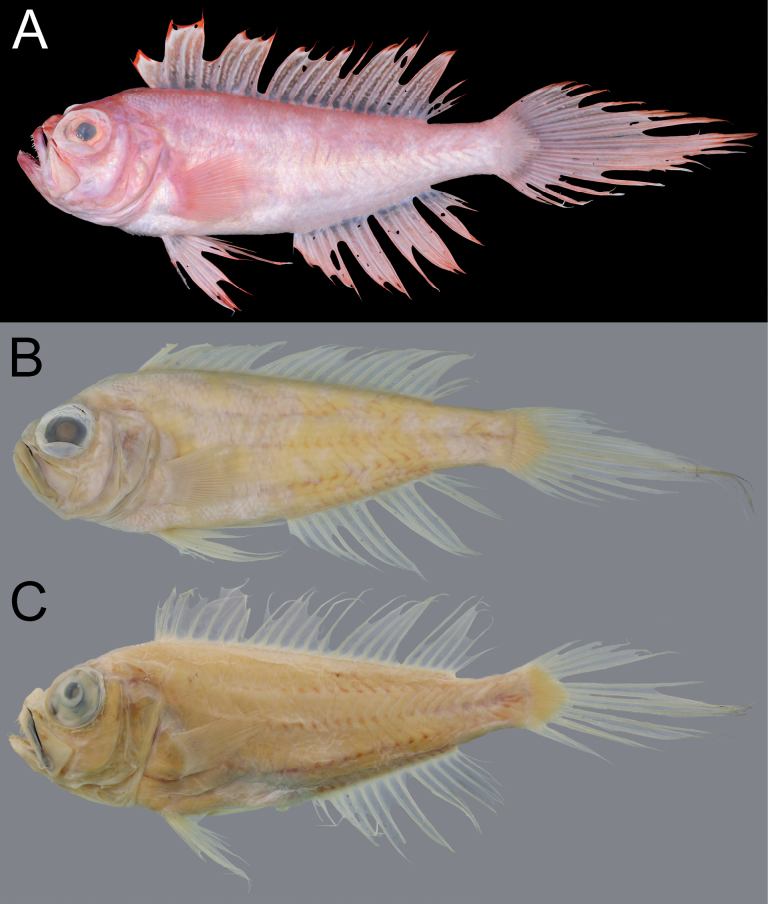
*Owstonia smithvanizi* sp. nov. A. Fresh specimen; B, C. Preserved coloration; A, B. NMMB-P30830, 194.1 mm SL, holotype; C. NMMB-P26744, 153.0 mm SL, paratype. Photographed by K. Koeda (A) and Y.-C. Hsu (B, C).

Fig. 1; Tables 1, 2 New English name: Smith-Vaniz’s Bandfish New Chinese name. 史密氏歐氏鰧

Owstonia psilos (non Smith-Vaniz & Johnson): Jhan and Ho 2019: 982, unnumbered figure (in part, misidentification; NMMB-P30830).

##### Type material.

***Holotype*** • NMMB-P30830, 194.1 mm SL; South China Sea, TAIWAN, Pingtung, Dong-gang fishing port; ca 22°22'22"N, 120°27'34"E; bottom trawl; H.-C. Ho leg. at a landing ground; 14 January 2019. ***Paratype*** • NMMB-P26744, 135.0 mm SL; same locality (landing ground) as holotype; H.-C. Ho leg., 23 June 2017.

##### Diagnosis.

A new species of *Owstonia* differing from its congeners in having the following combination of characters: lateral-line of type 3, extending beyond dorsal-fin origin but not forming loop, and no contact with posttemporal; dorsal-fin rays III, 21; anal-fin rays I, 14; gill rakers on outer face of first arch 15–16 + 29–31 = 45–46; oblique body scale rows 49–50; cheek scale rows ca 4–6; vertebrae 11 + 17 = 28; anal-fin pterygiophores anterior to 1^st^ haemal spine 2; preopercular spines 12–14; head length 27.6–28.3% SL; dorsal-fin base length 60.4–62.4% SL; anal-fin base length 30.7–31.9% SL; eye diameter 11.3–12.9% SL; premaxillary stripe present; and distal part of dorsal fin with very faint strip between third spine and second soft ray or entirely pale.

##### Description.

Meristic and morphometric data are provided in Tables 1, 2. Data of holotype are provided first, with those of paratype in parentheses whenever different.

**Table 1.** Meristic characters of *Owstonia smithvanizi* sp. nov. and compared to the type series of *O. rhamma* Smith-Vaniz & Johnson, 2016. Paired characters are presented as left/right whenever available. ^R^ denotes right side.

	*O. smithvanizi* sp. nov.		*O. rhamma*	
	This study		Smith-Vaniz and Johnson (2016)	
	Holotype	Paratype	Holotype	Non-type
Dorsal-fin rays	III, 21	III, 21	III, 21	
Pectoral-fin rays	21/21	21/21	19	21/23
Anal-fin rays	I, 14	I, 14	I, 14	
Vertebrae	11 + 17 = 28	11 + 17 = 28	11 + 17 = 28	
Anal-fin pterygiophores anterior to 1^st^ haemal spine	2	2	2	
Cheek-scale rows	ca 4^R^	ca 6	2 or 3	–
Oblique body scale rows	50/49	N/A	ca 50	–
Lateral-line terminus total dorsal-fin rays	24^th^/23^rd^	24^th^	22^nd^/23^rd^	19^th^
Gill rakers	16 + 29 = 45	15 + 31 = 46	16 + 29 = 45	15 + 30 = 45
Pseudobranchial filaments	31	30	–	–
Preoperclular spine	14/12	12/13	5–6	–
Premaxillary teeth	19/20	15/16	16/22	14
Inner premaxillary teeth	1/1	1/1	2	0
Lateral dentary teeth	14/14	14/13	11/12	11
Symphyseal teeth	4/4	5/4	4	3
Inner symphyseal teeth	1/2	1/1	2/1	1

**Table 2.** Morphometric characters of *Owstonia smithvanizi* sp. nov. and compared to the type series of *O. rhamma* Smith-Vaniz & Johnson, 2016.

	*O. smithvanizi* sp. nov.		*O. rhamma*	
	This study		Smith-Vaniz and Johnson (2016)	
	Holotype	Paratype	Holotype	Non-type
Standard length (mm)	194.1	135.0	102	50
% Standard length				
Head length	28.3	27.6	33.0	33.8
Predorsal length	25.9	28.3	27.2	27.9
Preanal length	55.4	59.5	56.7	55.0
Dorsal-fin base length	62.4	60.4	63.5	63.6
Dorsal-fin height	21.3	22.0	–	–
Anal-fin base length	31.9	30.7	35.3	32.9
Anal-fin height	21.8	21.7	–	–
Pectoral-fin length	20.8	22.2	–	–
Pelvic-spine length	N/A	14.1	–	–
Pelvic-fin length	29.9	25.7	27.9	31.3
Caudal-fin length	57.9	47.9	44.1	27.6
Body depth at anal-fin origin	28.5	27.4	28.7	29.3
Upper-jaw length	15.9	16.4	16.3	18.7
Upper-jaw depth	7.8	7.5	7.3	8.6
Eye diameter	11.3	12.9	13.5	16.4
Interorbital width	5.8	5.7	–	–
Caudal-peduncle length	15.8	15.0	–	–
Caudal-peduncle depth	11.6	10.6	–	–

Dorsal-fin rays III, 21; pectoral-fin rays 21/21; pelvic-fin rays I, 5/I, 5; anal-fin rays I, 14; principal caudal-fin rays 8 + 7, uppermost and lowermost rays unbranched; procurrent caudal-fin rays 4 and 3 on upper and lower lobes, respectively. Gill rakers on outer face of first gill arch 16 + 29 = 45 (15 + 31 = 46); pseudobranchial filaments 31. Oblique body scale rows 50/49; cheek scale rows ca 4 (right side)/ ca 6. Vertebrae 11 + 17 = 28; anal-fin pterygiophores anterior to 1^st^ haemal spine 2. Spines on preopercle 14/12 (12/13). Premaxillary teeth 19/20 (15/16); inner premaxillary teeth 1/1; lateral dentary teeth 14/14 (14/13); symphyseal teeth 4/4 (5/4); inner symphyseal teeth 1/2 (1/1).

Body slender, depth at anal-fin origin 3.5 (3.7 mm) in SL; both dorsal and ventral profiles of body straight, slightly tapering to caudal fin. Head large, length 3.5 (3.6) in SL; anterior profile of head rounded, gently curved to dorsal-fin origin. Eyes large, eye diameter 2.5 (2.1) in HL. Two nostrils, with anterior one forming tube and short flap; posterior one oval, without flap, situated immediately in front of eye. Preoprecle and opercle covered by skin; posterior margin of preopercle with weak serration.

Mouth lower in position, slightly oblique. Jaws terminated, with lower jaw slightly protruding before upper jaw. Upper-jaw length 1.8 (1.7) in HL, its end reaching to vertical through middle of eye; posterodorsal tip of ascending premaxillary processes with three pairs of papillae, with middle pair larger than rest (holotype), or with four larger papillae associated with smaller ones (paratype). Supramaxilla absent. Premaxilla with single row of canine teeth; teeth gradually smaller posteriorly; teeth slightly recurved and pointed or blunt. Dentary with single row of canine teeth; those on symphyseal slightly larger than others; some lateral teeth slightly enlarged anteriorly; teeth slightly recurved and pointed or blunt. Vomer and palatine without teeth.

Body scales cycloid and deciduous. Scales absent on isthmus, gular, both jaws, and interorbital space. Lateral line of type 3 (*sensu*Smith-Vaniz and Johnson 2016); its anterior end not forming complete loop; several scales originate from posttemporal but not connected with main branch. Lateral-line terminus below 24^th^/23^th^ (24^th^/24^th^) total dorsal-fin ray.

Dorsal fin with long base, originated at vertical through upper end of gill slit; its distal margin nearly straight, with slight elevation on soft rays; no distinct notch between spines and soft rays. Pectoral-fin tip rounded; its length 1.4 (1.2) in HL; its origin at same horizontal through lower margin of eye; its tip reaching to vertical through anal-fin origin. Pelvic fin elongated, its length 0.9 (1.1) in HL; its origin below 3^rd^ dorsal-fin spine; its tip reaching to 3^rd^ total anal-fin ray (holotype) or anus (paratype) when adpressed. Anal-fin base moderately long, its posterior end slightly anterior to that of dorsal fin; its origin below 9^th^ dorsal-fin soft ray; fin rays gradually longer posteriorly; its distal margin nearly straight. Caudal fin pointed; its length 0.5 (0.6) in HL. Caudal peduncle broad, its length and depth 1.8 and 2.4 (2.6) in HL, respectively.

##### Coloration.

When fresh (Fig. 1A), body, head, and all fins pink, with abdomen paler. Distal margin of dorsal, pelvic, anal, and caudal fins red. Dorsal-fin membranes with white vermicular pattern. When preserved (Fig. 1B, C), body uniformly pale. Distal part of dorsal fin with very faint strip between third spine and second soft ray (holotype) or pale (paratype). Membrane between premaxilla and maxilla uniformly black.

##### Etymology.

We are pleased to name this species after the late Dr William (Bill) F. Smith-Vaniz for his great contribution to our knowledge of cepolids and for his generous assistance in our previous paper.

##### Remarks.

The left-side cheek of holotype has only two rows of scales, and not extending to near preopercle, while the right side has four rows of scales extending to near preopercle. It is very likely the left side was damaged due to bottom trawl.

#### ﻿ Owstonia doryptera

Taxon classificationAnimaliaTeleosteiCepolidae

(Fowler, 1934)

6BE000F9-3148-5001-91B3-C1B006C6F108

**Figure 2.**
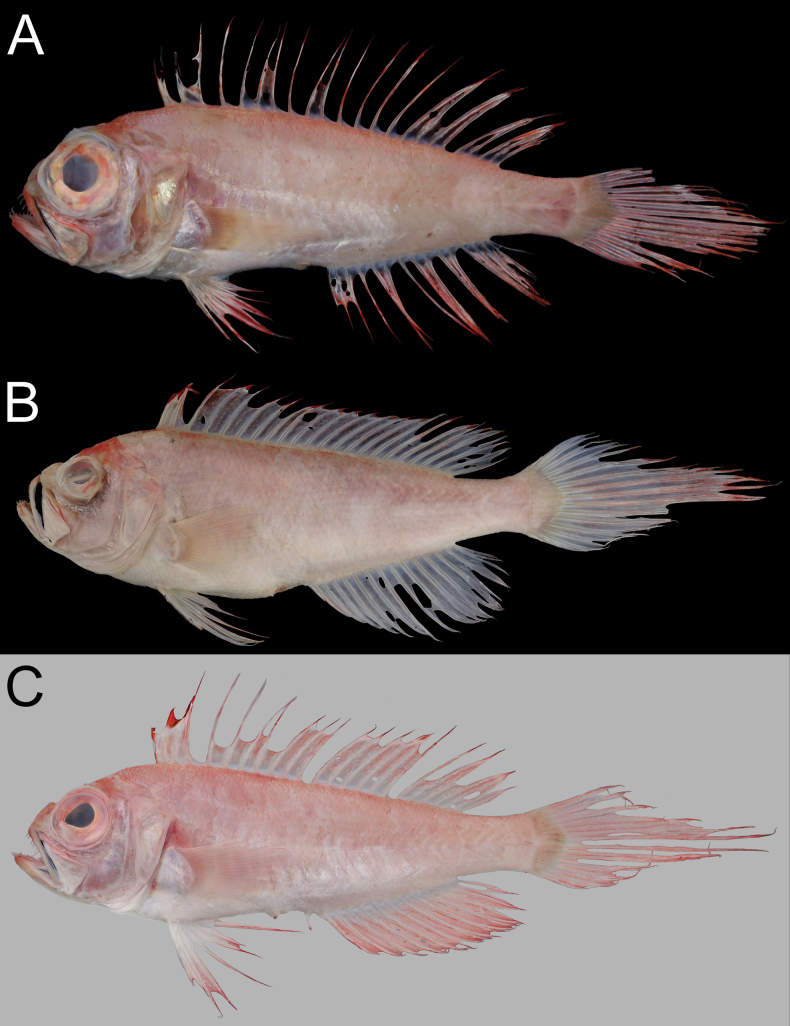
Fresh coloration of *Owstonia doryptera* (Fowler, 1934). A. NMMB-P42299, 109.1 mm SL; B. NMMB-P42267, 296.1 mm SL; C. NMMB-P42300, 143.0 mm SL. Photographed by Y.-C. Hsu.

**Figure 3.**
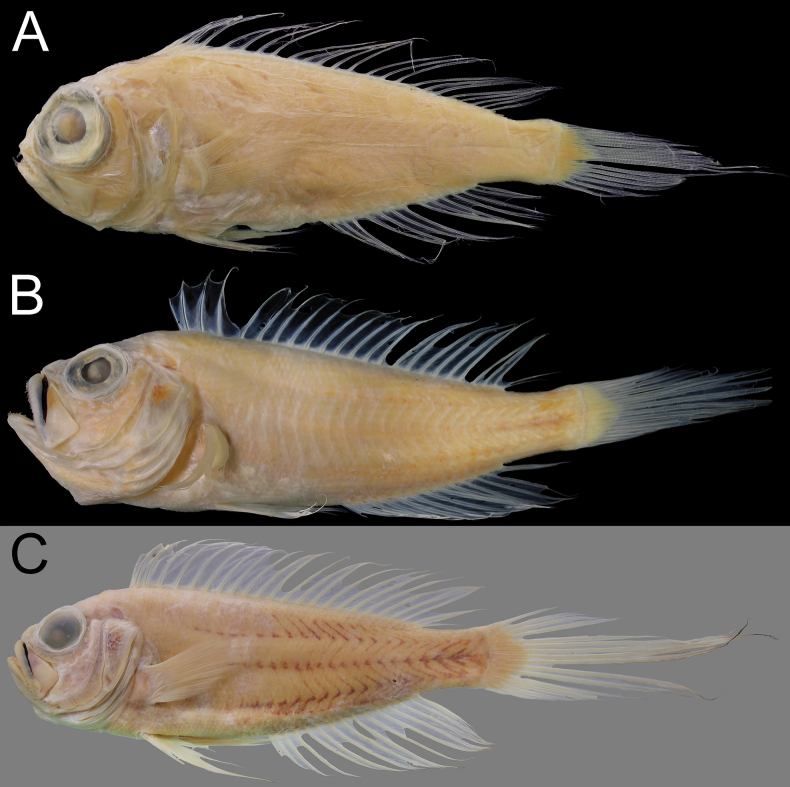
Preserved coloration of *Owstonia doryptera* (Fowler, 1934). A. NMMB-P36133, 89.7 mm SL; B. NMMB-P38895, 159.9 mm SL; C. NMMB-P32884, 200.2 mm SL. Photographed by Y.-C. Hsu.

**Figure 4.**
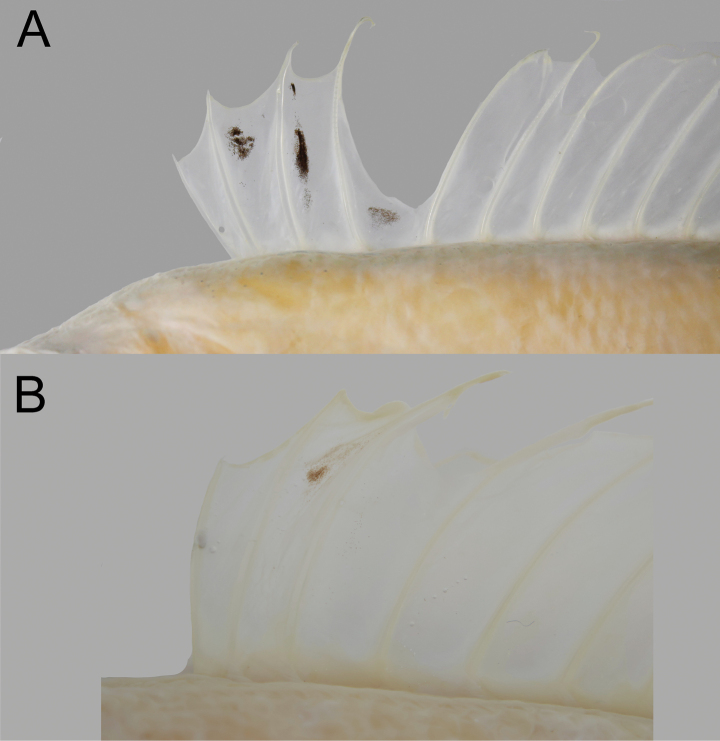
Coloration of anterior dorsal fin of *Owstonia doryptera* (Fowler, 1934), showing variations on black pigmentations. A. NMMB-P38895, 159.9 mm SL; B. NMMB-P32884, 200.2 mm SL. Photographed by Y.-C. Hsu. Anterior to left. Figure not to scale.

Figs 2, 3, 4; Tables 3, 4 English name: Spear-finned Bandfish New Chinese name: 矛鰭歐氏鰧

Loxopseudochromis doryptera Fowler, 1934: 354, fig. 106 (type locality: off northern Mindanao Island, Philippines, depth 175 fathoms [= 320 m]. Lectotype: USNM 93166).
Owstonia doryptera (Fowler 1934). Smith-Vaniz and Johnson 2016: 45 (redescription and lectotype designation). Liao et al. 2022:127 (listed in comparative material).
Owstonia dorypterus (Fowler 1934). Imai 2000: 625 (listed, South China Sea). Smith-Vaniz 2001: 3332 (listed). Liao et al. 2009: 522 (compared to the new species described).
Owstonia psilos (non Smith-Vaniz and Johnson). Jhan and Ho 2019:982, unnumbered figure (in part, misidentification; NMMB-P30640).

##### Specimens examined.

All collected from off Dong-gang fishing port (ca 22°22'22"N, 120°27'34"E), Pingtung, southwestern Taiwan • NMMB-P001317, 86.6 mm SL, 31 May 1985, coll. H.-C. Chang • NMMB-P30640, 158.3 mm SL, 10 September 2018, coll. K. Koeda and Y. Hibino • NMMB-P31226, 108.9 mm SL, 29 July 2018, coll. H.-C. Ho • NMMB-P32875, 108.4 mm SL • NMMB-P32884, 200.2 mm SL, 5 April 2019, coll. H.-C. Ho • NMMB-P33730, 2 specimens, 90.1–119.3 mm SL, 22 February 2020, coll. H.-C. Ho • NMMB-P34605, 233.7 mm SL, 30 July 2020, coll. H.-C. Ho • NMMB-P34901, 86.1 mm SL, 11 August 2020, coll. H.-C. Ho and C.-N. Tang • NMMB-P36076, 80.6 mm SL, 16 September 2020, coll. Y. Su, T.-K. Chou and N.-S. Leung • NMMB-P36133, 89.7 mm SL, 14 July 2021, coll. Y. Su, Y.-H. Kuo, and L. C. Halasan • NMMB-P38848, 2 specimens, 131.3–140.4 mm SL, 28 May 2022, coll. Y. Su • NMMB-P38895, 159.9 mm SL, 6 January 2023, coll. Y. Su, R.-Y. Hung, and Y.-C. Fan • NMMB-P42298, 78.3 mm SL, 9 May 2024, coll. Y. Su and Y.-C. Hsu • NMMB-P42267, 296.1 mm SL, 11 February 2025, coll. K.-H. Wu • NMMB-P42299, 109.1 mm SL, 8 March 2025, coll. K.-H. Wu • NMMB-P42300, 143.0 mm SL, 26 September 2024, coll. Y. Su and Y.-C. Hsu • NMMB-P42301, 105.2 mm SL, 2 April 2025, coll. H.-C. Ho and J.-Y. Chiang.

##### Description of Taiwanese specimens.

Meristic and morphometric data are provided in Tables 3, 4.

**Table 3.** Meristic characters of *Owstonia doryptera* (Fowler, 1934) and compared to type series. Paired characters are presented as left/right whenever available.

	This study	Smith-Vaniz and Johnson (2016)
	*n* = 18	All types (*n* = 2)
Dorsal-fin rays	III, 21 (rarely 20)	III, 21
Pectoral-fin rays	20–22/20–22	21–22
Anal-fin rays	I, 14	I, 14
Vertebrae	11 + 17 = 28	11 + 17 = 28
Anal-fin pterygiophores anterior to 1^st^ haemal spine	2	2
Cheek scale rows	4–6/4–6	5–6
Oblique body scale rows	39–56/39–59	ca 45
Lateral-line terminus total dorsal-fin rays	18^th^–24^th^/19^th^–24^th^	15^th^ or 17^th^
Gill rakers	15–17 + 28–31 = 43–48	16 + 26–28 = 42–44
Pseudobranchial filaments	24–43	–
Preoperclular spine	11–19/7–16	12–14
Premaxillary teeth	14–24/13–25	16/18
Inner premaxillary teeth	0–1/0–1	0
Lateral dentary teeth	12–19/12–18	9
Symphyseal teeth	3–5/3–5	5 or 6
Inner symphyseal teeth	0–6/0–6	1

**Table 4.** Morphometric characters of *Owstonia doryptera* (Fowler, 1934) and compared to type series.

	This study		Smith–Vaniz and Johnson (2016)	
	*n* = 18		Lectotype	Paralectotype
Standard length (mm)	78.3–296.1		65	56
	Mean (range)	SD	–	–
% Standard length			–	–
Head length	30.3 (26.9–34.0)	2.0	–	–
Predorsal length	28.0 (25.3–30.6)	1.8	30.7	34.6
Preanal length	56.8 (53.9–60.6)	1.9	59.4	58.6
Dorsal-fin base length	62.8 (59.1–65.1)	1.4	62.3	65.2
Dorsal-fin height	21.5 (19.1–23.7)	1.1	–	–
Anal-fin base length	30.8 (28.3–33.0)	1.4	30.4	31.1
Anal-fin height	23.0 (19.9–27.6)	2.0	–	–
Pectoral-fin length	22.0 (18.5–25.5)	2.0	–	–
Pelvic-spine length	14.9 (12.9–26.6)	1.2	–	–
Pelvic-fin length	26.9 (21.6–32.4)	2.4	25.7	25.4
Caudal-fin length	46.7 (39.5–60.1)	6.0	40.6	42.5
Body depth at anal-fin origin	27.1 (25.4–28.5)	1.0	27.6	27.5
Upper-jaw length	17.0 (14.3–18.6)	1.2	19.3	19.2
Upper-jaw depth	7.5 (6.7–8.1)	0.4	7.7	8.2
Eye diameter	14.0 (9.8–16.9)	2.2	16.0	17.3
Interorbital width	5.3 (4.6–6.1)	0.4	–	–
Caudal-peduncle length	15.9 (14.0–19.0)	1.3	–	–
Caudal-peduncle depth	10.7 (9.5–12.1)	0.6	–	–

Dorsal-fin rays III, 21 (one specimen with 20); pectoral-fin rays 20–22/20–22; pelvic-fin rays I, 5/I, 5; anal-fin rays I, 14; principal caudal-fin rays 8 + 7, uppermost and lowermost rays unbranched; procurrent caudal-fin rays 3–4 and 3–5 on upper and lower lobes, respectively. Gill rakers on outer face of first gill arch 15–17 + 28–31 = 43–48 (total); pseudobranchial filaments 24–43. Oblique body scale rows 39–56/39–59; cheek scale rows 4–6/4–6. Vertebrae 11 + 17 = 28 (*n* = 15); anal-fin pterygiophores anterior to 1^st^ haemal spine 2 (*n* = 15). Spines on preopercle 11–19/7–16. Premaxillary teeth 14–24/13–25; inner premaxillary teeth 0–1/0–1; lateral dentary teeth 12–19/12–18; symphyseal teeth 3–5/3–5; inner symphyseal teeth 0–6/0–6.

Body stout, depth at anal-fin origin 3.5–3.9 in SL; both dorsal and ventral profiles of body straight, slightly tapering to caudal fin. Head large, length 2.9–3.7 in SL; anterior profile of head rounded, gently curved to dorsal-fin origin. Eyes large, eye diameter 1.9–2.8 in HL. Two nostrils, with anterior one forming tube and short flap; posterior one oval, without flap, situated immediately in front of eye. Preoprecle and opercle covered by skin; posterior margin of preopercle with enlarged spines, those on vertical portions forming serrations.

Mouth lower in position, slightly oblique. Jaws terminal, with lower jaw slightly protruding before upper jaw. Upper-jaw length 1.6–1.9 in HL, its end reaching vertical through middle of eye to slightly anterior to vertical through posterior margin of eye; posterodorsal tip of ascending premaxillary processes with four larger papillae associated with smaller ones. Supramaxilla absent. Premaxilla with single row of canine teeth; teeth gradually smaller posteriorly; teeth slightly recurved and pointed or blunt. Dentary with single row of canine teeth; those on symphyseal slightly larger than others; some anterior lateral teeth slightly enlarged or not; teeth slightly recurved and pointed or blunt. Vomer and palatine without teeth.

Body scales cycloid and deciduous. Scales absent on isthmus, gular, both jaws, and interorbital space. Lateral line type 3; its anterior end forming complete loop. Lateral line terminates below 18^th^–24^th^/19^th^–24^th^ total dorsal-fin ray.

Dorsal fin with long base, originating at vertical through upper end of gill slit; its distal nearly straight, with slight elevation on soft rays; no distinct notch between spines and soft rays. Pectoral-fin tip rounded; its length 1.2–1.5 in HL; its origin slightly above horizontal through lower margin of eye; its posterior end variable, reaching from anterior to vertical through anal-fin origin to reaching beyond vertical through anal-fin origin. Pelvic fin elongated, length 0.9–1.4 in HL; its origin below third dorsal-fin spine; its tip also variable reaching from anus to 4^th^ total anal-fin ray when adpressed (correlated with specimen size). Anal-fin base moderately long, its posterior end slightly anterior to that of dorsal fin; its origin below 11^th^ or 12^th^ total dorsal-fin ray; fin rays gradually longer posteriorly; its distal margin nearly straight. Caudal fin pointed; its length 0.5–0.8 in HL. Caudal peduncle broad, depth 2.4–3.4 in HL and length 1.5–2.3 in HL.

##### Coloration.

When fresh (Fig. 2), body, pectoral fin, and dorsal-, pelvic-, anal-, and caudal-fin membranes pink, with dorsum, top of head, and distal margin of dorsal, pelvic, anal, and caudal fins red. Dorsal-, pelvic-, anal-, and caudal-fin rays paler than their fin membranes. When preserved (Fig. 3), body and fins uniformly pale, except for NMMB-P42299, which has dusky middle caudal-fin rays. Black stripe or blotch on dorsal fin variable (Fig. 4), from uniformly pale to black between second spine and first soft ray or between second and third spines. Membrane between premaxilla and maxilla uniformly black. Inner face of preopercle slightly dusky.

##### Distribution.

Previously known only from the type series collected from northern Mindanao Island, Philippines at depth 320 m. Our specimens represent the northernmost record of this species. This species appears to be rather commonly collected by bottom trawl in southern Taiwan. The estimated depth of catch is about 200–400 m.

##### Remarks.

Our specimens are identified as *O. doryptera* in having: anal-fin rays I, 14; cheek scales present; lateral line of type 3, forming complete loop in front of dorsal fin (Smith-Vaniz and Johnson 2016). Compared to the type specimens, several major differences were found between our specimens and the type series. Our specimens possess a more posterior end of lateral line (ending at 18^th^–24^th^ total dorsal-fin ray, vs 15^th^ or 17^th^ in *O. doryptera*; Smith-Vaniz and Johnson 2016; Table 3); and more lateral dentary teeth (12–19 vs 9). As other characters, such as the number of oblique scale rows and premaxillary teeth display considerable variation, and some traits (e.g. pseudobranchial filaments, lateral dentary teeth, end of pelvic fin, head length, and caudal-fin length) are growth dependent, these differences are interpreted as intraspecific variation.

#### ﻿ Owstonia cf. aurora

Taxon classificationAnimaliaTeleosteiCepolidae

41C6A332-A16F-5415-B1C2-0AC6FCFF6005

**Figure 5.**
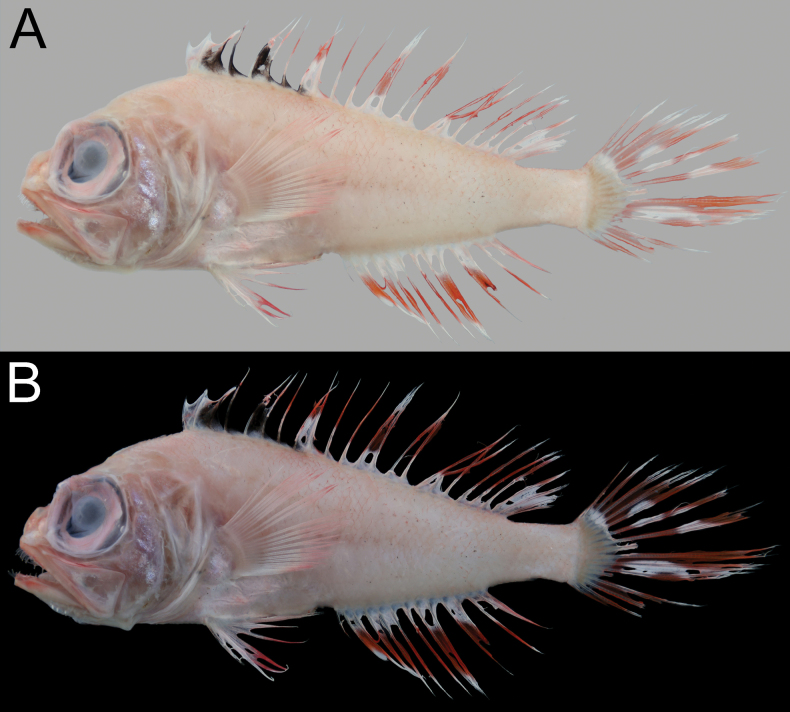
Fresh coloration of Owstonia cf. aurora, NMMB-P42302, 73.7 mm SL. A. On gray background; B. On black background. Photographed by Y.-C. Hsu.

**Figure 6.**
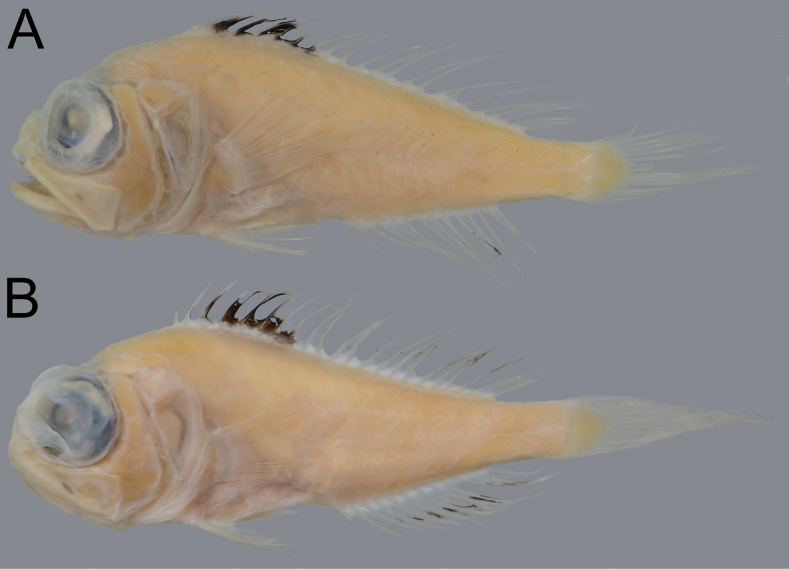
Preserved coloration of Owstonia cf. aurora. A. NMMB-P42302, 73.7 mm SL; B. NMMB-P29294, 76.7 mm SL.

Figs 5, 6; Tables 5, 6

Owstonia totomiensis (non Tanaka): Jhan and Ho in Koeda and Ho 2019: 984 (in part, misidentification; NMMB-P29294).

##### Specimen examined.

All collected from off Dong-gang fishing port (ca 22°22'22"N, 120°27'34"E), Pingtung, southwestern Taiwan • NMMB-P29294, 76.6 mm SL, 3 April 2018, collected by K. Koeda • NMMB-P42302, 73.7 mm SL, collected by Y. Su, Y.-C. Hsu, H.-C. Ho.

##### Description of Taiwanese specimens.

Meristic and morphometric data are provided in Tables 5, 6.

**Table 5.** Meristic characters of Owstonia cf. aurora and compared to type series of *O. aurora* Liao, Rodolfo & Shao, 2022. Paired characters are presented as left/right whenever available.

	O. cf. aurora		*O. aurora*	
	This study		Liao et al. (2022)	
	NMMB-P29294	NMMB-P42302	Holotype	Paratype (*n* = 2)
Dorsal-fin rays	III, 20	III, 21	III, 21	
Pectoral-fin rays	22/22	22/23	20–22	
Anal-fin rays	I, 13	I, 13	I, 14	
Vertebrae	11 + 17 = 28	11 + 17 = 28	11 + 17 = 28	
Anal-fin pterygiophores anterior to 1^st^ haemal spine	2	2	2	
Cheek-scale rows	9	7	8/8	7–8/8
Oblique body scale rows	46/47	ca 57/ca 50	58	54–56
Lateral-line terminus total dorsal-fin rays	11^th^/N/A	N/A	20^th^ to 25^th^*	
Gill rakers	12 + 24 = 36	13 + 24 = 37	11 + 24 = 35	12 + 26 = 38
Pseudobranchial filaments	26	25	–	–
Preoperclular spine	0	0	0	
Premaxillary teeth	25/21	19/21	14–15	14–16
Inner premaxillary teeth	0	0	0	
Lateral dentary teeth	9/10	13/14	13	13–16
Symphyseal teeth	6/5	4/4	3–5	
Inner symphyseal teeth	1/1	1/1	1–2	

**Table 6.** Morphometric characters of Owstonia cf. aurora and compared to type series of *O. aurora* Liao, Rodolfo & Shao, 2022.

	O. cf. aurora		*O. aurora*	
	This study		Liao et al. (2022)	
	NMMB-P29294	NMMB-P42302	Holotype	Paratype (n = 2)
Standard length (mm)	76.6	73.7	74.9	69.8–88.0
% Standard length				
Head length	35.6	36.6	36.2	34.1–34.4
Predorsal length	35.7	34.7	34.7	33.8–34.5
Preanal length	57.3	57.4	57.5	57.0–61.9
Dorsal-fin base length	59.6	63.5	61.9	59.0–60.2
Dorsal-fin height	N/A	22.2	–	–
Anal-fin base length	28.2	28.0	31.0	23.6–26.1
Anal-fin height	22.9	22.4	–	–
Pectoral-fin length	25.5	24.7	19.8	19.5–21.1
Pelvic-spine length	12.9	N/A	–	–
Pelvic-fin length	22.3	24.0	21.2	19.8–20.5
Caudal-fin length	42.1	N/A	35.8	34.1
Body depth at anal-fin origin	24.9	26.1	35.4	28.7–35.2
Upper-jaw length	21.5	22.2	16.8	15.0–16.5
Upper-jaw depth	9.3	9.5	8.9	8.0–8.6
Eye diameter	16.8	16.8	14.7	14.8–16.4
Interorbital width	4.9	5.3	5.2	4.9–5.2
Caudal-peduncle length	16.2	17.4	15.5	13.6–14.4
Caudal-peduncle depth	9.5	9.4	10.9	9.8–11.0

Dorsal-fin rays III, 20–21; pectoral-fin rays 22/22–23; pelvic-fin rays I, 5/I, 5; anal-fin rays I, 13; principal caudal-fin rays 8 + 7, uppermost and lowermost rays unbranched; procurrent caudal-fin rays 3 on both upper and lower lobes. Gill rakers on outer face of first gill arch 12–13 + 24 = 36–37(total); pseudobranchial filaments 25–26. Oblique body scale rows 46–ca 57/47–ca 50; cheek scale rows 7–9/9. Vertebrae 11 + 17 = 28; anal-fin pterygiophores anterior to 1^st^ haemal spine 2. Spines on preopercle 0/0. Premaxillary teeth 19–25/21; inner premaxillary teeth 0/0; lateral dentary teeth 9–13/10–14; symphyseal teeth 4–6/4–5; inner symphyseal teeth 1/1.

Body slender, depth at anal-fin origin 3.8–4.0 in SL; both dorsal and ventral profiles of body straight, slightly tapering to caudal fin. Head large, length 2.7–2.8 in SL; anterior profile of head rounded, gently curved to dorsal-fin origin. Eyes large, eye diameter 2.1–2.2 in HL. Two nostrils, with anterior one forming tube and short flap; posterior one oval, without flap, situated immediately in front of eye. Preoprecle and opercle covered by skin; posterior margin of preopercle smooth

Mouth lower in position, slightly oblique. Jaws terminated, with lower jaw slightly protruding before upper jaw. Upper-jaw length 1.6–1.7 in HL, its end reaching to vertical through middle of eye; posterodorsal tip of ascending premaxillary processes with two or three pairs of small papillae. Supramaxilla absent. Premaxilla with single row of canine teeth; teeth gradually smaller posteriorly; teeth slightly recurved and pointed or blunt. Dentary with single row of canine teeth; those on symphyseal slightly larger than others; some lateral teeth slightly enlarged anteriorly; teeth slightly recurved and pointed or blunt. Vomer and palatine without teeth.

Body scales cycloid and deciduous. Scales absent on isthmus, gular, both jaws, and interorbital space. Lateral line type 1; its origin from posttemporal and along dorsal-fin base; not extending beyond dorsal-fin origin. Lateral line terminates below 11^th^ total dorsal-fin ray (*n* = 1).

Dorsal fin with long base, originated at vertical through upper end of gill slit; its distal nearly straight, with slight elevation on soft rays; no distinct notch between spines and soft rays. Pectoral-fin tip rounded; its length 1.4–1.5 in HL; its origin at same horizontal through lower margin of eye; its tip reaching to anal-fin origin. Pelvic fin elongated, its length 1.4–1.5 in HL; its origin below 3^rd^ dorsal-fin spine; its tip reaching to anus when adpressed. Anal-fin base moderately long, its posterior end slightly anterior to that of dorsal fin; its origin below 12^th^ total dorsal-fin ray; fin rays gradually longer posteriorly; its distal margin nearly straight. Caudal fin rounded, slightly pointed; its length 0.8 in HL (*n* = 1). Caudal peduncle broad, length and depth 2.1–2.2 and 3.6–3.9 in HL, respectively.

##### Coloration.

When fresh (Fig. 4), body, pectoral fin, with dorsum, top of head, dorsal- and anal-fin soft rays, distal margin of pelvic-fin soft rays, and caudal fin red. Distal and proximal margins of dorsal and anal fins with white band. Caudal fin with ca three large white blotches. When preserved (Fig. 5), body uniformly pale. Black stripe or blotch on dorsal fin between second spine and sixth soft ray. Membrane between premaxilla and maxilla pale or slightly dusky (with small dusky blotch on right side of NMMB-P42302). Inner face of preopercle slightly dusky. Anal fin with black spots (spots fewer and more faint in NMMB-P42302).

##### Remarks.

Jhan and Ho (2019) reported three specimens of *O. totomiensis* (NMMB-P23318, NMMB-P23838, and NMMB-P29294), of which the latter is here identified as O. cf. aurora, and the other two specimens are identified as *O. totomiensis*.

Liao et al. (2022) stated that their *O. aurora* has lateral line ends below 17^th^–22^nd^ dorsal-fin soft ray; however, it is apparently an error as they provided 21 dorsal-fin soft rays throughout the article. See Discussion for further comparison.

## ﻿Discussion

### ﻿Establishment of new species

Among congeners, *Owstonia smithvanizi* sp. nov. is similar to *O. doryptera*, *O. maccullochi* Whitley, 1934, *O. nudibucca* Smith-Vaniz & Johnson, 2016, *O. rhamma* Smith-Vaniz & Johnson, 2016, and *O. totomiensis*, in having lateral line of type 3 (Smith-Vaniz and Johnson 2016). The presence of cheek scales differentiates *O. smithvanizi* from *O. nudibucca* and *O. totomiensis* (Smith-Vaniz and Johnson 2016; this study).

According to Smith-Vaniz and Johnson (2016), among the rest species, *O. smithvanizi* is most similar to *O. rhamma* in having: anal-fin rays I, 14 (vs I, 15–16, rarely I, 14 in *O. maccullochi*; Smith-Vaniz and Johnson 2016); gill rakers 15–16 + 29–31 = 45–46 (vs 17–19 + 31–35 = 49–54 in *O. maccullochi*) lateral-line extending before dorsal-fin origin and not forming complete loop (vs forming complete loop in *O. doryptera*). However, *O. smithvanizi* possess more preopercular spines (12–14 vs 5–6 in *O. rhamma*; Smith-Vaniz and Johnson 2016; Table 1); more cheek-scale rows (ca 4–6 vs 2–3); shorter head (27.6–28.3% SL vs 33.0–33.8% SL; Table 2); shorter dorsal-fin base (60.4–62.4% SL vs 63.5–63.6% SL); shorter anal-fin base (30.7–31.9% SL vs 32.9 –35.3% SL); and smaller eye (11.3–12.9% SL vs 13.5–16.4% SL).

Although previously reported as *Owstonia psilos* Smith-Vaniz & Johnson, 2016, the new species differs from *O. psilos* in having: lateral line of type 3 (vs type 1 in *O. psilos*; Smith-Vaniz and Johnson 2016); oblique body scale rows 49–50 (vs ca 32); preopercular spines 12–14 (vs 0); premaxillary stripe present (vs absent); head length 27.6–28.3% SL (vs 31.0–35.8% SL); caudal-fin length 47.9–57.9% SL (vs 35.0–39.0); body depth at anal-fin origin 27.4–28.5% SL (vs 22.4–26.0% SL); upper-jaw length 15.9–16.4% SL (vs 18.2–20.8% SL); and upper-jaw depth 7.5–7.8% SL (vs 8.4–9.6% SL).

### ﻿Differences between *O. doryptera* and *O. rhamma*

Smith-Vaniz and Johnson (2016) proposed several characters to differentiate *O. dorptera* and *O. rhamma*, including: the stripe between 2^nd^ dorsal-fin spine and 1^st^ soft ray (present in *O. doryptera* vs absent in *O. rhamma*); lateral-line pattern anterior to dorsal fin (complete loop vs incomplete loop); cheek scale rows (5–6 vs 2 or 3); size of lateral dentary canines (not enlarged, vs enlarged); and end of pelvic fin when adpressed (extending to anal-fin origin vs to base of 2^nd^ or 4^th^ anal-fin soft ray); and number of inner teeth on premaxilla (0 vs 2). However, the coloration on dorsal fin is variable (Fig. 4). Moreover, the end of the pelvic fin is size correlated (reaching to anus and becoming posteriorly as growing). Therefore, the only reliable character to separate these two species is the lateral-line pattern anterior to dorsal fin and cheek scale rows.

### ﻿Differences between O. cf. aurora and *O. aurora*

The present specimens mostly resemble *Owstonia aurora* Liao, Rodolfo & Shao, 2022, originally described from the Philippines and later recorded from off Dong-sha Island, South China Sea (Liao et al. 2022; Ng et al. 2024). Both sharing: lateral line of type 1, originating from posttemporal and along dorsal-fin base; vertebrae 11 + 17 = 28; lower margin of preopercle smooth, without any spines or serrations; black stripe on dorsal fin between second spine and sixth soft ray; and either similar or overlapping counts in gill rakers, oblique body scale rows, and cheek scale rows (Liao et al. 2022).

However, our specimens differ from the type series of *O. aurora* in having more premaxillary teeth (19–25 vs 14–16; Liao et al. 2022; Table 5), anterior positioned lateral-line end (11^th^ total dorsal-fin ray vs 20^th^ to 25^th^; *n* = 1), longer pectoral fin (24.7–25.5% SL vs 19.5–21.1% SL; Table 6), longer caudal fin (42.1% SL vs 34.1–35.8% SL), longer upper jaw (21.5–22.2% SL vs 15.0–16.8% SL), and anal-fin coloration (with black spots or stripe vs pale). Although our specimens probably represent a new species, we refrain from naming them due to the damaged lateral-line termination and similar coloration with that of *O. aurora*.

According to Smith-Vaniz and Johnson (2016), the present specimens key out to *O. psilos*. However, our specimens differ from *O. psilos* in having: oblique body scale rows 46–ca 57 (vs ca 32 in *O. psilos*; Smith-Vaniz and Johnson 2016); cheek scale rows 7–9 (vs ca 5–6); and lateral line terminus below 11^th^ total dorsal-fin ray (vs 17^th^–23^rd^).

## ﻿Checklist and comments on species of *Owstonia* from Taiwan

Table 7 listed species of *Owstonia* recorded from Taiwan known to date. Nine species have been recorded: *O. auroraO. doryptera*, *O. grammodon*, *O. kamoharai*, *O. smithvanizi* sp. nov., *O. taeniosoma*, *O. tosaensis*, *O. totomiensis*, and O. cf. aurora (Shen 1993; Shen and Wu 2011; Endo et al. 2015; Smith-Vaniz and Johnson 2016; Jhan and Ho 2019; Ng et al. 2024; this study). The record of *O. psilos* by Jhan and Ho (2019) is now re-identified as either *O. doryptera* or *O. smithvanizi*, and one of the specimens of *O. totomiensis* by Jhan and Ho (2019) is now re-identified as O. cf. aurora.

**Table 7.** Checklist of species of *Owstonia* Tanaka, 1908 recorded from Taiwan historically.

Species	Chinese name	Reference
*O. aurora* Liao, Reyes & Shao, 2022	奧羅拉歐氏鰧	Ng et al. 2024
*O. doryptera* (Fowler, 1934)	矛鰭歐氏鰧	Jhan and Ho 2019 (partly as *O. psilos*); This study
*O. grammodon* (Fowler, 1934)	粒牙歐氏鰧	Endo et al. 2015; Smith-Vaniz and Johnson 2016; this study
*O. kamoharai* Endo, Liao & Matsuura, 2015	蒲原氏歐氏鰧	Chiang et al 2014 (as *O.* sp.); Jhan and Ho 2019
*O. smithvanizi* sp. nov.	史密氏歐氏鰧	Jhan and Ho 2019 (partly as *O. psilos*); this study
*O. taeniosoma* (Kamohara, 1935)	長身歐氏鰧	Shen 1993; Shen and Wu 2011; Jhan and Ho 2019
*O. tosaensis* Kamohara, 1934	土佐歐氏鰧	Shen 1993; Shen and Wu 2011; Smith-Vaniz and Johnson 2016
*O. totomiensis* Tanaka, 1908	遠江歐氏鰧	Shen 1993; Jhan and Ho 2019; this study
O. cf. aurora		Jhan and Ho 2019 (partly as *O. totomiensis*); this study

Smith-Vaniz and Johnson (2016) noted the Taiwanese specimens of *O. grammodon* differ from that collected from the type locality (Sulawesi). The Taiwanese specimens possess 12 + 16 = 28 vertebrae (vs 11 + 17 =28 in Sulawesi specimens; Smith-Vaniz and Johnson 2016), anal-fin pterygiophores anterior to 1^st^ haemal spine 4 (vs 3), and premaxillary teeth 33–34 (vs 36−39). Examination of additional specimens also revealed same counts of vertebrae and anal-fin pterygiophores; however, the premaxillary teeth ranges from 30 to 37. As suggested by Smith-Vaniz and Johnson (2016), more studies are needed to determine whether the Taiwanese specimens represent a new species.

Although Jhan and Ho (2019) provided the vertebral formula as 12 + 15 = 27 for *O. kamoharai*, a re-examination of one voucher specimen, NMMB-P27521 reveals 11 + 17 = 28.

### ﻿Comparative materials

*Owstonia grammodon*: NMMB-P17742, 96 mm SL, and NMMB-P20801, 125 mm SL, off Daxi Fishing Port (ca 24°53'37"N, 121°55'26"E), Yilan, northeastern Taiwan, 12 November 2012, collected by H.-C. Ho. *O. kamoharai*: NMMB-P27521, 159 mm SL, off Dong-gang fishing port, Pingtung, southwestern Taiwan, 1 November 2017, collected by K. Koeda. NMMB-P34034, 100 mm SL, off Dong-sha Islands, South China Sea, collected by H.-C. Ho. NMMB-P39099, 291 mm SL, off Cheng-gong fishing port (ca 23°05'53.4"N, 121°22'46.0"E), Taitung, eastern Taiwan, 10 June 2023, collected by J.-F. Huang. *O. totomiensis*: NMMB-P23318, 121 mm SL, off Dong-gang fishing port, 2 April 2016, collected by H.-C. Ho. NMMB-P23838, 94 mm SL, off Dong-gang fishing port, 20 February 2016, collected by H.-C. Ho. NMMB-P32877, 121.5 mm SL, off Dong-gang fishing port, 1 April 2019, collected by H.-C. Ho.

## Supplementary Material

XML Treatment for Owstonia smithvanizi

XML Treatment for Owstonia doryptera

XML Treatment for Owstonia cf. aurora

## ﻿Additional information

### Use of AI

ChatGPT was used to correct grammar errors and wordings. All sentences were originally written by the authors.

### Author ORCIDs

Yo Su https://orcid.org/0000-0002-3576-9229

Hsuan-Ching Ho https://orcid.org/0000-0003-1154-601X

### Data availability

All of the data that support the findings of this study are available in the main text.
